# Enhancing feed fermentation in rice straw basal diets using leaf protein concentrate as a novel ruminant supplement derived from *Indigofera zollingeriana*

**DOI:** 10.14202/vetworld.2024.2077-2087

**Published:** 2024-09-15

**Authors:** Wisri Puastuti, Dwi Yulistiani, Tuti Haryati, Susana Insusila Watining Rakhmani, Yeni Widiawati, Diana Andrianita Kusumaningrum, Elizabeth Wina, Anuraga Jayanegara, Markus Anda

**Affiliations:** 1 Research Center for Animal Husbandry, Research Organization for Agriculture and Food, National Research and Innovation Agency, Indonesia; 2 Indonesian Research Institute for Animal Production, Bogor, Indonesia; 3 Faculty of Animal Science, IPB University, Indonesia; 4 Research Center for Horticulture, Research Organization for Agriculture and Food, National Research and Innovation Agency, Indonesia

**Keywords:** distilled water solvent, *Indigofera*, leaf protein concentrate, rice straw, rumen fermentation

## Abstract

**Background and Aim:**

Rice straw, a widely available agricultural byproduct globally, has significant potential as a basal diet for livestock. The major challenge lies in obtaining high-protein foliage that can be easily extracted using natural water rather than chemical solvents. This study aimed to assess the ability of distilled water to extract protein concentrate from *Indigofera* leaves (*Indigofera zollingeriana* Miq.) and to evaluate its effectiveness in enhancing rumen feed fermentation and digestibility in low-quality rice straw basal diets.

**Materials and Methods:**

The study was conducted in two experimental series. Experiment 1 was designed to explore the ability of distilled water to extract protein concentrate from fresh and dry *Indigofera* leaves by comparing it with the 0.1 N NaOH standard solvent. Experiment 2 focused on the *in vitro* digestibility of protein concentrates extracted from fresh *Indigofera* leaves based on optimal findings from experiment 1. Five treatments consisting of 0.5% and 1.0% protein concentrate and two extractants (distilled water and 0.1 N NaOH) were used to extract protein from *Indigofera* leaves. These extracts were then added to rice straw-based diets. Rice straw without supplements was used as a control. The treatments were arranged using a randomized complete design with five replicates.

**Results:**

The results of experiment 1 showed that distilled water was superior to 0.1 N NaOH for extracting protein concentrate from fresh *Indigofera* leaves, as revealed by higher dry matter, protein yield, total amino acids (AA), and total essential AA (EAA) production. For *in vitro* experiment 2, supplementation with distilled water-extracted protein concentrates successfully increased rumen fermentation and digestibility in rice straw basal diets, as indicated by higher gas production, total volatile fatty acid, and microbial protein levels compared with 0.1 N NaOH.

**Conclusion:**

Findings from this study confirm that *Indigofera* leaf protein concentrate offers a new alternative for enhancing rumen feed fermentation and the digestibility of low-quality rice straw diets. This study implies that it is an easy, cost-effective, and environmentally friendly approach, particularly beneficial for smallholders, to extract protein concentrate from fresh *Indigofera* leaves using distilled water and use it to enhance the quality of rice straw for ruminant feed. The limitation of this study is that the *Indigofera* supplement was established using *in vitro* digestibility under controlled laboratory conditions, which does not reflect real rumen conditions. Therefore, further studies using *in vivo* digestibility in ruminant animals are required to confirm the ability of the protein extracted from *indigofera* to enhance rumen feed fermentation in low-protein basal diets.

## Introduction

Rice straw is an agricultural byproduct with the potential to serve as fodder for ruminants due to its widespread availability after rice harvesting in many areas, low cost, and practicality as a feed source [1, 2]. Annually, an estimated 800–1000 million tons of rice straw are produced worldwide, with approximately 600–800 million tons originating from Asia (https://www.irri.org/rice-straw-management). However, feeding ruminants with pure rice straw is of insufficient quality to satisfy ruminant requirements, notably during periods of rapid growth and early lactation. This is attributed to the lower dry matter (DM) intake and protein content of rice straw (4.0%–4.7% crude protein [CP]), along with elevated levels of silica and lignin, resulting in low nutrient digestibility (<50%) [3]. Therefore, pre-treatment of rice straw is crucial for enhancing DM intake, nutrient digestibility, and overall animal performance [2]. The challenge lies in seeking forage with high protein content and digestibility that can serve as a supplement to enhance the nutritional value of rice straw. Legume forage is a valuable source of dietary protein that thrives in tropical climates, making it available locally and widely. One prominent legume forage is *Indigofera* spp., which is known for its leaves, which contain high-quality proteins with a complete amino acid (AA) composition [4, 5]. *Indigofera* *zollingeriana* Miq. leaves are used as a substitute for soybean meal, which is known as the best high-quality protein source, in animal rations [6]. Both *Indigofera* and soybean meal contain complete AA content, including histidine, threonine, arginine, tyrosine, methionine, phenylalanine, valine, lysine, leucine, and isoleucine [5]. The complete of AA content in *Indigofera* can be directly utilized by rumen microbes if the protein is obtained in the form of a leaf protein concentrate (LPC) with high digestibility [5]. LPC is a protein derived from fibrous components of green leafy biomass [7]. LPC can be used as a protein source for animal feed with low fiber content and exhibits superior nutritional quality [8]. The advantages of LPC extend to its excellent water solubility, high surface activity [9, 10], and higher digestibility than leaf meal [11].

A recent review by Heppner and Livney [12] described 45 types of green leaves as sources of protein concentrate using organic, alkali, acidic, and salting solvents. They concluded that green plant leaves represent a promising sustainable source of LPC for animals. At present, limited information is available on the extraction and precipitation process using distilled water for protein extraction from fresh foliage leaves. A previous study by Tripathi *et al*. [13] demonstrated that distilled water is a feasible method for extracting protein from *Girardinia heterophylla* leaves. The use of distilled water, which is environmentally sound and economically beneficial, requires further studies to determine its ability to extract protein from the leaves of different legumes. Here, we used distilled water to extract protein concentrate from fresh leaves of *Indigofera* and compared the results with those obtained using leaf protein extracted using NaOH as the standard solvent. The obtained LPC is used as a feed supplement for ruminants to provide distilled water as an alternative solvent for leaf protein production.

This study aimed to assess the ability of distilled water, representing natural conditions, to extract protein concentrate from *Indigofera* leaves (*I. zollingeriana* Miq.), and to evaluate its effectiveness in enhancing rumen feed fermentation and digestibility in low-quality rice straw basal diets.

## Materials and Methods

### Ethical approval

The experiments (protocol number 1806.202.051B/H1/APBN 2019) were conducted under ambient laboratory conditions. The use of animals was approved by the Institutional Animal Care and Uses Committee (IACUC) of the Ministry of Agriculture Indonesia (Approval number: Balitbangtan/Balitnak/Rm/02/2019).

### Study period and location

The study was conducted from June to November-2019 at the Indonesian Research Institute for Animal Production, Indonesian Agency of Agriculture Research and Development, Ministry of Agriculture, Indonesia.

### Experiment 1. Extracting protein concentrate from *Indigofera leaves*

*I. zollingeriana* leaves were harvested from experimental stations at the Indonesian Research Institute for Animal Production. *I. zollingeriana*, a superior tree legume, was endorsed by the decree of the Indonesian Minister of Agriculture (number: 19 KPTS/KB. 020/2/2019). The foliage of *Indigofera* (*I. zollingeriana* Miq.) at 3 months of regrowth consisted of the young stem and leaf, and the tip was chopped to approximately 3 cm in size and divided into two parts. One part was directly used for the fresh extraction process, and the other part was dried in the oven for 48 h at 60°C to achieve 90% DM, finely ground, and used for the dry leaf extraction process. The study was set up as a 2 × 2 factorial experiment with five replicates and arranged in a randomized complete design. The first factor was *Indigofera* leaves at two states (ID = dry and IF = fresh), and the second factor was solvent consisting of two types (W = distilled water and N = 0.1 N NaOH), resulting in the combination treatment of *Indigofera* dry leaves extracted with water solvent (IDW), *Indigofera* dry leaves extracted with 0.1 N NaOH solvent (IDN), *Indigofera* fresh leaves extracted with water solvent (IFW), and *Indigofera* fresh leaves extracted with 0.1 N NaOH solvent (IFN).

Freshly chopped and ground dry leaves of *Indigofera* were extracted using distilled water and 0.1 N NaOH solvent. The extraction process was conducted by modifying the method reported by Coldebella *et al*. [8] and. Klupšaitė and Juodeikienė [14]. Briefly, distilled water or 0.1 N NaOH was added to chopped fresh *Indigofera* leaves (ratio 1: 3 w/v), and then, each mixture was blended and filtered using a cotton cloth to obtain leaf juice. For dried *Indigofera* leaves, the solvent ratio was 1:5 w/v to ensure a water content similar to that of fresh leaves. The dry leaves were mixed with solvent and stirred for 30 min, filtered using a cotton cloth, and the juice was collected. The pH of the juice was adjusted by adding 0.1 N HCl to the juice until it reached the pH isoelectric point (pHip), which accelerates protein deposition. To determine the pHip, each juice was transferred into duplicates of five tubes containing 10 mL each to span the original pH between 2 and 8 by adding 0.1 N HCl or NaOH 0.1 N solution. This mixture was then kept for 24 h in a refrigerator at temperatures 4°C. Next, each tube was centrifuged at 1,700× *g* for 10 min using the Suprena 21 A high-speed refrigeration centrifuge (Tomy Kogyo Co. Ltd., Japan), and the supernatant were discarded. The tubes containing the precipitate were dried in an oven at 60°C and then weighed to determine the dry weight. The pH and dry weight were regressed by plotting the data on a graph and fitting the regression equation to determine the pHip. The maximum point of the regression equation indicates the pHip at which the highest concentration of protein concentrate is deposited.

The pHip point was used as a reference for producing protein concentrates by adjusting the pH of the juice to the isoelectric point. Four types of protein concentrate were produced from *Indigofera* leaves: IFWIFN, IDW, and IDN. Each extraction process was performed in five replicates. Protein concentrates from *Indigofera* were analyzed for DM, CP, and AA content. The AA content of each LPC was determined using five replicates.

The mass yield of protein concentrate (DM yield), the extraction of protein yield (protein yield), and the increase in CP content from the original ingredients were calculated using the formula of Coldebella *et al*. [8] as follows:

- MYPC = (PCM/ILM) × 100
  Here, MYPC = Mass yield of the protein concentrate.
  PCM = Protein concentrate mass (g) on a dry basis
  ILM = *Indigofera* leaf mass at the beginning of extraction (g) on dry
- EPY = (CPPC/CPBE) × 100
  Here, EPY = The extraction of protein yield
  CPPC= CP of the protein concentrate mass (g) on a dry basis
  CPBE = CP present in the leaf at the beginning of extraction (g) on a dry basis
- Increase in CP content (%) = ([%CP content of mass yield- % CP content in *Indigofera* leaf at the beginning of extraction]/%CP content of *Indigofera* leaf at the beginning of extraction) × 100%

Data were analyzed using one way analysis of variance for a randomized complete design using SAS 9.0 software [15].

### Experiment 2. Supplementation of rice straw with *Indigofera* LPC

#### In vitro digestibility

The findings from experiment 1 indicate that the protein concentrate extracted from fresh *Indigofera* leaves is superior to that extracted from dry leaves. Therefore, fresh leaves were used for *in vitro* digestibility in experiment 2. Protein concentrates extracted from fresh *Indigofera* leaves using distilled water (W) and 0.1 N NaOH (N) were then supplemented with rice straw basal diets. The rice straw basal diet was composed of 50% rice straw and 50% concentrate and was described to have a CP content of 12.75% and a metabolizable energy of 1944 Kcal/kg. The concentrate consisted of rice bran, cassava waste, palm kernel cake, pollard, molasses, soybean meal, dried distilled grain soluble, urea, salt, and minerals.

Five diet treatments consisted of a rice straw basal diet as a control, and rice straw supplemented with *Indigofera* protein concentrate at concentrations of 0.5% and 1.0% were extracted using two extractants (distilled water and 0.1 N NaOH). The five treatments were designated as Control, IFW05, IFW1, IFN05, and IFN1.

Where IF = *Indigofera* protein concentrate, W = distilled water, and N = 0.1 N NaOH. Arabic suffix notations of 05 and 1 indicate 0.5% and 1.0% protein concentrate, respectively. The experimental design was randomized with five treatments and five replicates.

The percentage of protein concentrates was calculated using the DM diet. Diets were evaluated using *in vitro* digestibility methods reported by Menke and Steingass [16] and Blummel *et al*. [17]. Rumen fluid was obtained from a cattle slaughterhouse in Bogor, Indonesia. Truly degradable fermented substrates (*in vitro* true DM degradability (IVTDMD) were determined by incubating samples for 48 h. A 500 mg sample was transferred to a serum bottle, added to 40 mL of medium, and incubated in a shaker water bath at 39°C for 48 h. After incubation, gas volumes were measured at 2, 4, 8, 12, 24, and 48 h. After incubation, 20 mL of supernatant was collected using a syringe to analyze volatile fatty acids (VFA), ruminal ammonia nitrogen (NH_3_-N), protozoa, bacteria, and protein rumen microbial production. The residue in the serum bottle was transferred into a 600 mL spoutless beaker. The bottle was washed with 70 mL of NDS solution. The procedure of Van Soest *et al*. [18] was then applied by refluxing the incubation residue for 1.0 h and filtering the undigested matter on pre-tared filter crucibles.

### Chemical analysis

Chemical analysis of the DM, OM, and CP feeds was performed according to the AOAC procedure [19]. Ruminal NH_3_ concentrations were measured using the Conway microdiffusion technique [20]. The total and partial VFA concentrations were evaluated by gas chromatography (Chrompack CP-9002, Chrompack, Inc., Raritan, New Jersey, USA). The microbial population, referred to as the number of protozoa, was determined using a hemocytometer, and the bacterial population was recorded using the roll-tube method of Ogimoto and Imai [21]. The production of microbial proteins was measured using the Lowry method [22]. CO_2_ and CH_4_ gas production composition was analyzed according to the Tjandraatmadja [23] procedure. Data were analyzed by one way analysis of variance using SAS v 9.0 software (SAS Institute, Inc. Cary, NC. USA) [15]. Differences among means were compared using Duncan’s multiple range test at a probability of 5%.

## Results

### Experiment 1. Protein concentrate production

**Figure-1:**
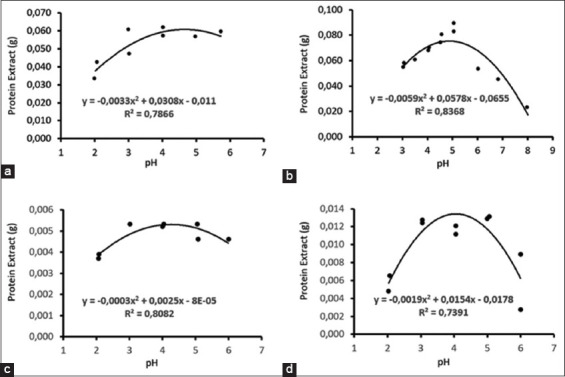
The pH isoelectric point of the *Indigofera* protein concentrate in distilled water and 0.1 N NaOH: (a) Fresh *Indigofera* leaves in distilled water, (b) Fresh *Indigofera* leaves in 0.1 N NaOH, (c) Dry *Indigofera* leaves in distilled water, and (d) Dry *Indigofera* leaves in 0.1 N NaOH.

The pHip of *Indigofera* in distilled water were 4.4 and 4.2 for fresh and dry leaves, respectively (Figure-1). In addition, the pHip values of fresh and dry leaves in 0.1 N NaOH were 4.6 and 4.0, respectively. The pHip values were considerably higher in fresh than in dried *Indigofera* leaves. The higher pHip values for fresh leaves than for dry leaves in the two solvents indicate that the drying process decreased protein solubility. The same pHip was observed for distilled water and 0.1 N NaOH, indicating that the two solvents have comparable ability to extract protein concentrate from *Indigofera* leaves.

The mass yields of crude protein concentrate (MYPC) and the extracted protein yield (EPY) from fresh and dry *Indigofera* leaves using distilled water and NaOH are presented in Table-1. MYPC and EPY levels were affected (p < 0.05) by the interaction between leaf form and solvent type. Fresh leaves extracted with water (IFW) produced higher MYPC and EPY than those extracted with NaOH, whereas MYPC and EPY extracted from dry leaves were not affected by the presence of different solvents (IDW and IDN). The higher MYPC in fresh than in dry *Indigofera* leaves may be due to the more soluble protein in the natural state of fresh *Indigofera* leaves, which is easier to extract than the corresponding protein that undergoes drying in dried leaves.

**Table-1:** Dry matter and crude protein yield of the *Indigofera* leaf protein concentrate.

Leaf condition	Solvent	Protein	

		MYPC (%)	EPY (%DM)
Fresh leaf (IF)	Water (W)	29.70^a^ ± 3.25	39.83^a^ ± 4.36
	0.1 N NaOH (N)	12.92^b^ ± 3.50	18.10^b^ ± 4.90
Dry leaf (ID)	Water (W)	0.67^c^ ± 0.17	0.68^c^ ± 0.17
	0.1 N NaOH (N)	0.74^c^ ± 0.20	0.69^c^ ± 0.18
p-values			
Main factor	Leaf condition	<0.0001	<0.0001
	Solvent	<0.0001	<0.0001
Interaction Leaf×Solvent		<0.0001	<0.0001

MYPC=Mass yield of protein concentration, EPY=Extraction of protein yield, values with different letters in the same column indicate significant differences (p < 0.05)

The CP content of the *Indigofera* LPC extracted with either distilled water or 0.1 N NaOH exhibited a higher value in fresh leaves than in dry leaves (Table-2). Compared with the control treatment, the CP content of fresh leaves increased by 34.15% and 40.14% in distilled water and 0.1 N NaOH extractants, respectively. The corresponding values for dry leaves were 1.27 and 2.44%, respectively.

**Table-2:** Crude protein content of *Indigofera* leaves and increased leaf protein concentrate extracted with distilled water and 0.1 N NaOH.

Parameter	Control	*Indigofera* leaf protein concentrate			

		IFW	IFN	IDW	IDN
Crude protein (CP) content (g/kg)	307.2	412.1	430.5	311.1	314.7
Increased CP content (%)	0	34.15	40.14	1.27	2.44

Control: leaf meal (not extracted), IFW=Fresh *Indigofera* leaves extracted with distilled water, IFN=Fresh *Indigofera* leaves extracted with 0.1 N NaOH, IDW=Dried *Indigofera* leaves extracted using distilled water, IDN=Dried *Indigofera* leaves extracted with 0.1 N NaOH. The data were obtained from the analysis of 5 replicates that were composited

The AA composition of LPCs extracted from fresh and dried *Indigofera* leaves using distilled water and NaOH is presented in Table-3. The AA content of LPC extracted from fresh leaves (IFW) in distilled water resulted in the highest total AA and EAA contents of approximately 337.8 and 183.2 g/kg, respectively. This represents an increase of about 1.34–1.40 times from the *Indigofera* leaf meal (control, not extracted). Moreover, extraction of fresh *Indigofera* leaves using distilled water with acid precipitation yielded a protein concentrate of superior quality.

**Table-3:** Amino acid composition of protein concentrate extracted from *Indigofera* leaves using distilled water and 0.1 N NaOH.

Amino acid (%)	*Indigofera* leaf protein concentrate				

	Control	IFW	IFN	IDW	IDN
Non-essential					
Aspartic	2.32	3.13	2.16	1.80	2.16
Serine	1.34	0.97	0.04	0.59	1.85
Glutamic	2.10	3.96	4.37	2.07	0.71
Glysine	1.87	2.27	0.01	1.02	0.01
Alanine	1.22	2.43	0.33	0.96	0.05
Proline	1.74	1.36	1.63	0.76	0.01
Cysteine	0.31	0.16	0.19	0.03	0.05
Tyrosine	1.28	1.18	1.10	0.69	0.73
Essential					
Methionine	0.43	0.76	0.46	0.29	0.03
Arginine	1.88	1.87	1.96	0.97	0.04
Threonine	0.14	1.31	0.26	0.73	2.02
Histidine	0.84	0.89	0.82	0.41	0.62
Phenilalanine	2.07	2.37	1.89	1.04	1.30
Lysine	1.59	2.25	2.19	0.95	0.02
Valine	1.84	2.80	2.41	1.26	0.28
Leusine	2.28	3.69	2.88	1.64	1.99
Isoleusine	1.60	2.38	1.77	1.06	1.28
Total AA	24.85	33.78	24.47	16.27	13.15
Total EAA	12.67	18.32	14.64	8.354	7.58

Control=*Indigofera* leaf meal, IFW=Fresh *Indigofera* leaves extracted with distilled water, IFN=Fresh *Indigofera* leaves extracted with 0.1 N NaOH, IDW=Dried *Indigofera* leaves extracted with distilled water, IDN=Dried *Indigofera* leaves extracted with 0.1 N NaOH. The data were obtained from the analysis of 5 replicates that were composited, AA=Amino acids, EAA=Essential amino acids

In contrast to fresh leaves, the total AA content from dry leaf extraction in distilled water and NaOH solvents decreased from 24.85% to 16.27% and 13.15%, respectively. Similarly, the EAA content decreased from 12.67% to 8.35% and 7.58% after dry leaf extraction. Compared with leaf meal (control), the content of three-branch chain AAs (BCAAs) in the protein concentrate obtained from fresh leaves (IFW) increased by 52.2, 61.8, and 48.8% for valine, leucine, and isoleucine, respectively. In contrast, there was no increase in BCAA content in dry *Indigofera* leaves extracted using distilled water or 0.1 N NaOH.

### Experiment 2. Supplementation of rice straw with *Indigofera* LPC

#### In vitro digestibility

The IVTDMD and *in vitro* true organic matter degradability (IVTOMD) of IFW1, IFN1, and IFW05 were not significantly different compared with the control treatment (p > 0.05), but they were significantly higher than that of the IFN05 treatment (p < 0.05) (Table-4). The IVTDMD and IVTOMD values of the ratios ranged from 65.98% to 69.87% and 66.83% to 71.47%, respectively. Improved dry matter degradability and organic matter degradability were associated with fermentable proteins derived from protein concentrate supplementation in rice straw-based rations to provide BCAA (valine, isoleucine, and leucine) that stimulate *in vitro* rumen fermentation.

**Table-4:** Dry matter and organic matter degradability in rations supplemented with leaf protein concentrate.

Variables	Ration treatment					p-value

	IFW1	IFN1	IFW05	IFN05	Control	
DMD (%)	68.26^a^ ± 0.80	68.45^a^ ± 1.04	69.87^a^ ± 0.77	65.98^b^ ± 0.96	68.18^a^ ± 1.65	0.0001
OMD (%)	68.45^a^ ± 1.16	68.00^a^ ± 0.55	71.47^a^ ± 1.74	66.83^b^ ± 1.09	67.22^a^ ± 1.03	0.0001

DMD=dry matter degradability, OMD=Organic matter degradability, IFW=Fresh *Indigofera* leaves extracted with distilled water, IFW1=A ration containing 1% concentration of IFW, IFN1=A ration containing 1% concentration of IFN, IFW05=A ration containing 0.5% concentration of IFW, IFN05=A ration containing 0.5% concentration of IFN, Control: *Indigofera* leaf meal (LPC not extracted); numbers with different letters in the same row indicate significant differences (p < 0.05)

### Rumen fermentation

The use of protein concentrates as supplements in the ration did not affect the ruminal pH, as revealed by the absence of differences between treatments and the control (Table-5). The rumen pH was varied from 6.75 to 6.83, which is the ideal condition for rumen. The protein concentrate was degraded in the rumen into NH_3_, which significantly increased the ruminal NH_3_ concentration. Supplementation of 1% LPC derived from freshly extracted leaves using distilled water (IFW1) and NaOH (IFN1 treatment) increased the protein content of the diet by 0.82% and 0.86%, respectively. The ruminal NH_3_ concentrations in IFW1 and IFN1 were not significantly different (16.19 ± 1.43 mM vs. 15.69 ± 0.85 mM), indicating that both solvents produced LPC with similar degradability in the rumen (p > 0.05). These NH_3_ concentrations were categorized as a high level. In contrast, supplementation with 0.5% protein concentrate did not affect the ruminal ammonia NH_3_ concentration.

**Table-5:** Mean pH, NH_3_, total gas, and CH_4_ production in ratios supplemented with leaf protein concentrate of *Indigofera*.

Variable	Ration treatment					p-value

	IFW1	IFN1	IFW05	IFN05	Control	
pH	6.75^a^ ± 0.06	6.80^a^ ± 0.08	6.78^a^ ± 0.10	6.83^a^ ± 0.10	6.83^a^ ± 0.10	0.6901
NH_3_ (mM)	16.19^a^ ± 1.43	15.69^a^ ± 0.85	12.06^b^ ± 0.83	12.88^b^ ± 0.75	12.50^b^ ± 0.61	0.0001
Total Gas (ml)	81.92^a^ ± 1.26	65.42^c^ ± 0.96	65.17^c^ ± 1.29	75.42^b^ ± 1.71	65.42^c^ ± 0.96	0.0001
CH_4_ (ml)	27.13^a^ ± 0.48	21.00^c^ ± 0.00	20.00^d^ ± 0.00	25.00^b^ ± 0.00	20.88^c^ ± 0.25	0.0001
CH_4_ (%)	33.11^a^ ± 0.39	32.11^b^ ± 0.47	30.70^c^ ± 0.61	33.16^a^ ± 0.74	31.91^b^ ± 0.42	0.0001

IFW=Fresh *Indigofera* leaves extracted with distilled water, IFW1=A ration containing 1% IFW, IFN1=A ration containing 1% IFN, IFW05=A ration containing 0.5% IFW, IFN05=A ration containing 0.5% IFN, C=Control, numbers with different letters in the same row indicate significant differences (p < 0.05)

Furthermore, supplementation with protein concentrates in the ration significantly increased total gas production (p < 0.05) (Table-5) with the highest value observed in the IFW1 ration. There was a positive correlation between total gas production (Y) and CH_4_ production (X) with equation Y = 2.45X + 14.73 (R² = 0.98), suggesting that CH_4_ plays a crucial role in total gas production. Gas production from feed fermentation is closely related to VFA production. Statistically, LPC supplementation did not significantly increase total VFA production (p > 0.05). However, there was a clear trend (p < 0.088) in the increase in total VFA content from 52.07 (control) to 56.78 mM and 69.44 mM in protein concentrate supplementation (extracted with distilled water) at 0.5% (IFW05) and 1.0% (IFW1) rations, respectively. On the other hand, protein-concentrate supplementation significantly decreased butyric/nC_4_ and valeric/nC_5_ acids (Table-6). The IFW1 ration produced the highest total VFA content (69.44 mM, marking a 33.56% increase compared with the control diet. In contrast, the IFN1, IFW05, and IFN05 diets produced lower total VFAs (49.01–56.78 mM), which were not significantly different from the control.

**Table-6:** *In vitro* rumen volatile fatty acid (VFA) concentrations of rations with protein concentrate supplement.

Variable	Ration treatment					p-value

	IFW1	IFN1	IFW05	IFN05	Control	
Total VFA content (mM)	69.44^a^ ± 9.14	49.23^a^ ± 9.99	56.78^a^ ± 3.99	49.13^a^ ± 13.37	52.07^a^ ± 13.82	0.0886
Proportion of partial VFA (%)						
Acetic, C_2_	52.54^ab^ ± 0.67	53.79^ab^ ± 4.72	52.25^ab^ ± 3.73	56.38^a^ ± 4.38	48.70^c^ ± 2.06	0.0804
Propionic, C_3_	26.35^a^ ± 0.68	25.16^a^ ± 3.03	22.84^a^ ± 1.82	24.95^a^ ± 2.52	25.79^a^ ± 1.63	0.2033
Iso-butyric, iC_4_	2.65^a^ ± 0.18	2.16^a^ ± 0.63	3.21^a^ ± 0.79	1.97^a^ ± 1.71	3.27^a^ ± 0.43	0.2031
Butyric, nC_4_	14.47^bc^ ± 0.70	15.33^abc^ ± 1.65	16.99^ab^ ± 0.99	14.12^c^ ± 1.84	17.59^a^ ± 1.48	0.0411
Iso-valeric, iC_5_	1.95^a^ ± 0.13	1.58^a^ ± 0.46	2.36^a^ ± 0.58	1.44^a^ ± 1.25	2.40^a^ ± 0.31	0.1986
Valeric, nC5	2.05^ab^ ± 0.11	1.51^bc^ ± 0.61	2.36^a^ ± 0.27	1.15^c^ ± 0.68	2.25^a^ ± 0.30	0.0073
C_2_ : C_3_	2.00^a^ ± 0.05	2.13^a^ ± 0.39	2.31^a^ ± 0.33	2.27^a^ ± 0.25	1.90^a^ ± 0.17	0.1972

IFW=Fresh *Indigofera* leaves extracted with distilled water, IFN=Fresh *Indigofera* leaves extracted with 0.1 N NaOH, IFW1=Control ration containing 1% of IFW, IFN1=Control ration containing 1% of IFN, IFW05=control ration containing 0.5% of IFW, IFN05=Control ration containing 0.5% of IFN, Control=rice straw basal ration, numbers with different letters in the same row indicate significant differences (p < 0.05)

The proportions of acetic (C_2_) and propionic (C_3_) acids, iso-butyric (iC_4_), and iso-valeric (iC_5_) acids, and C_2_:C_3_ ratios were similar (p > 0.05) due to the basal rations had similar NDF content (Table-6). Compared with the control, the effects of protein concentrate supplementation on butyric and valeric acid content varied (Table-6). For example, LPC supplementation decreased the proportion of nC_4_ from 17.59 % in the control to 14.47% in IFW1. LPC supplementation of IFW1 and IFW05 did not affect nC_5_, while supplementation of IFN1 and IFN05 supplementation reduced the proportion of nC_5_.

### Microbial protein synthesis

Protein concentrate supplementation in rations significantly increased (p < 0.05) the percentage of total bacteria, protozoa population, and microbial protein synthesis (Table-7). The interesting result is the higher-level protein concentrate supplementation (IFW1 and IFN1) significantly produced a low population of bacteria, while protozoa population significantly increased. Conversely, lower-level protein concentrate supplementation (IFW05 and IFN05) significantly increased the proportion of bacteria but significantly decreased the protozoal population. All protein concentrate supplementation rates did not significantly affect the microbial protein content. Although IFW1, IFN1, and IFN05 treatments increased by 18.9%, 11.40%, and 10.32%, respectively, compared with the control, they did not significantly differ.

**Table-7:** Total of bacteria, protozoa, and protein microbes in the ration supplemented with the protein concentrate of *Indigofera*.

Variable	Ration treatment					p-value

	IFW1	IFN1	IFW05	IFN05	Control	
Total bacteria* (×10^10^ cfu/mL)	2.54^e^ ± 0.14	3.05^d^ ± 0.20	4.40^b^ ± 0.09	4.81^a^ ± 0.39	3.47^c^ ± 0.06	0.0001
Protozoa* (×10^6^ cell/mL)	6.08^a^ ± 2.04	4.57^b^ ± 4.33	2.19^d^ ± 3.12	3.26^c^ ± 3.33	3.19^c^ ± 1.80	0.0001
Microbial protein (mg/100 mL)	160.88^a^ ± 3.75	150.67^ab^ ± 24.5	129.69^b^ ± 13.02	149.21^ab^ ± 16.24	135.25^ab^ ± 21.10	0.0087

IFW=Fresh *Indigofera* leaves extracted with distilled water, IFN=Fresh *Indigofera* leaves extracted with 0.1 N NaOH, IFW1=Control ration containing 1% of IFW, IFN1=Control ration containing 1% of IFN, IFW05=Control ration containing 0.5% of IFW, IFN05=Control ration containing 0.5% of IFN, Control=Rice straw basal ration,

* Statistical tests are based on logarithmically transformed data; numbers with different letters in the same row indicate significant differences (p < 0.05)

## Discussion

### Protein Concentrate production from *Indigofera* leaves

The CP content of the *Indigofera* leaves in this study was 307.6 g/kg (Table-2), which fell within the maximum range of CP content reported previously (223.0–311.0 g/kg) by Palupi *et al*. [5], Pudjihastutia *et al*. [24], and Kurniawan *et al*. [25]. The high CP content suggests that *Indigofera* is a promising alternative source of protein supplements for low-quality animal basal diets, such as rice straw. The findings confirm the efficacy of distilled water as a solvent for optimizing protein extraction from fresh *Indigofera* leaves compared with the chemical solvent 0.1 N NaOH. In large-scale protein production, using water as a solvent is a more accessible, cost-effective [26], practical, and environmentally sustainable approach for extracting LPC.

One study employed distilled water to extract LPC in Nettle (*G. heterophylla*) followed by a series of steps, including heating to 80°C for 8–10 min in a water bath to coagulate the protein, centrifugation at 10,624× *g* for 10 min, rinse with distilled water, and drying in an oven at 60°C for 30 min [13]. This method is more labor-intensive and time-consuming than the approach employed in this study. Our method involves the initial extraction of protein with distilled water from fresh *Indigofera* leaves followed by pH adjustment of the extracted juice to the isoelectric point (pHip) to precipitate the protein, thereby facilitating the process.

The average pHip of distilled water from fresh and dry *Indigofera* leaves was 4.3. At pHip, protein precipitates and clumps [27] to produce high LPC. The optimal pHip point for proteins generated from *Indigofera* was within the range of those from soybean and cassava leaves. Leaf protein was precipitated from the green juice of soybean leaves at pH 3.7 [28] and cassava leaves at pHip 4.0–5.0 [8, 29]. The protein structure and chemical characteristics of leaves affect the optimal pH for protein extraction [30] and the efficiency of extraction methods [31].

In fresh *Indigofera* leaves, water produced higher MYPC and EPY compared to those extracted with 0.1 N NaOH, whereas in dry leaves, there was no difference between the two solvents. This indicates that *Indigofera* leaves contain high levels of protein. According to Chen *et al*. [26], water extraction is typically used for proteins with high solubility and stability.

Extraction with 0.1 N NaOH should provide a high-pH environment and produce a higher LPC yield than water extraction at low pH. However, the current study obtained the opposite results, indicating that less MYPC and EPY were produced when using NaOH as a solvent compared to water extraction (Table-1). Higher LPC yields in water extraction could be affected by the nature of the protein in *Indigofera*, namely, its high solubility, which makes it easier to extract with water under natural conditions than the corresponding protein undergoing drying in dried leaves. Drying also caused chemical property changes in proteins and bound them to other matrices (i.e., fiber), as well as cell damage during the delivery of proteins to the extractant (water) [8]. The MYPC of *Indigofera* in the present study was higher than that of *G. heterophylla* leaves extracted with water, which produced 8 g of every 100 g of fresh leaves, as described by Tripathi *et al*. [13]. The CP content of LPCs is also affected by plant species. For example, LPCs derived from carrot, potato, and cassava leaves contained CP contents of 23%, 50%, 40%–45%, respectively [29, 32].

The increase in CP content in the protein concentrate extracted from fresh *Indigofera* leaves was lower than that reported by Yatno *et al*. [33] for Leucaena LPC, with a 94.3% increase in protein content. The higher protein extracted from Leucaena than from *Indigofera* could be attributed to the high pH employed during extraction (pH 8–10 using NaOH solvent) for leucaena. In the present study, however, the pH ranged from 2.0 to 8.0. Leaf protein extraction at high pH (7.0–8.0) was found to improve juice extraction and protein recovery. The use of alkali swollen the fibers in the material, making it easier for the protein to be extracted, in which protein becomes more soluble and cell wall destruction becomes more efficient [34]. The disparities in the increase in the CP content of LPC between our study and previous investigations may arise from variations in the extraction process, solvent, solid-to-solvent ratio, and pH during the extraction.

The use of distilled water to extract LPC from fresh *Indigofera* leaves is the first such study. Therefore, our findings offer a novel approach for protein extraction from *Indigofera*, a legume abundant in tropical regions. The extracted protein was used to enhance rumen fermentation in an *in vitro* digestibility experiment.

### *In vitro* digestibility of an *Indigofera* LPC in rice straw

Supplementation of *Indigofera* LPC to rice straw-based ration, serving as a source of fermentable protein that provides BCAA (valine, isoleucine, and leucine), has been found to stimulate *in vitro* rumen fermentation. The sufficiency of BCAA availability was revealed by the ideal rumen ecology, namely, the rumen pH (6.8) and NH_3_-N concentration (12.06–16.19 mM) to support rumen microbial activity (Table-5). According to Souza *et al*. [35], the normal rumen pH is in the range from 5.3 to 7.0 with an average of 6.1, while Wanapat *et al*. [36] suggested that the ideal rumen pH for fibrous feed digestion is in the range between 6.3 and 6.8.

In ruminant animals, only three BCAAs of the EAA, namely, valine, leucine, and isoleucine, are considered crucial. These BCAAs play a pivotal role in increasing protein synthesis in the rumen, thereby enhancing the population growth of ruminal microorganisms [37, 38].

In the present study, the IVTDMD and IVTOMD of rice straw-based rations supplemented with LPC extracted from fresh leaves (IFN and IFW) were higher (68.3%) than those reported in a previous study by Zhao *et al*. [39], where the in *vitro* DM digestibility (IVDMD) of rice straw-based rations supplemented with various doses of molasses as a sugar source ranged from 53.36% to 57.1%. Similarly, Yulistiani *et al*. [40] reported that IVDMD and IVOMD of rice straw basal feed with and without complete rumen modifier supplementation are in the range of 46.56%–59.12% and 50.33%–62.16%, respectively.

Supplementation with 1% protein concentrate derived from fresh leaf extraction (IFW1 and IFN1 treatments) significantly increased the ruminal NH_3_ concentration compared with the control diet. In contrast, supplementation with 0.5% protein concentrate did not affect the ruminal NH_3_ concentration. According to Paengkoum *et al*. [41], the ruminal NH_3_ concentrations needed by rumen microbes for maximum feed digestion were 5–20 mg/dL, which is equivalent to 3.57–14.28 mM. The high ruminal NH_3_ concentrations (15.69–16.19 mM) in IFW1 and IFN1 ration treatments were due to (i) protein concentrate supplementation obtained from precipitation of soluble protein from fresh *Indigofera* leaves that contain a high CP (412.1 and 430.5 g/kg) (Table-2) which is easily degraded by rumen microbes, and (ii) the low utilization of NH_3_ by rumen microbials for protein synthesis is attributed to the low bacteria population in IFW1 and IFN1 rations (Table-6).

The availability of carbohydrates, balance N, and energy in rumen are required to maximize the efficiency of microbial protein synthesis [42]. The lack of synchronization between energy and nitrogen can reduce microbial protein synthesis rates, resulting in the accumulation of NH_3_ [43]. In this study, rations were expressed in iso-energy, potentially leading to an excess of NH_3_ with the addition of LPC. Therefore, 1% leaf protein concentrate (IFW1) should be supplemented with carbohydrates to enhance microbial protein synthesis.

Gas production reflects rumen microbial activities, and the amount of energy produced during feed fermentation processes [43]. Supplementation with protein concentrates affected total gas production (p < 0.05), as shown in the IFW1 ration (Table-5). This indicates that supplementation of 1.0% LPC extracted with distilled water from fresh *Indigofera* leaves is effective for the fermentation process in the rumen, especially for fermenting fiber carbohydrates, as indicated by the increased production of methane gas (CH_4_). Enteric CH_4_ production depends on the fiber content in the ratio, which leads to longer rumen retention. Enteric methane emission has a positive correlation with fiber content but a negative correlation with dietary lipids [44]. In this study, the basal ratio of all treatments was similar; therefore, the difference in methane gas production could be due to different levels of protein concentrate supplementation (1.0 vs. 0.5%). In addition, the higher CH_4_ production in IFW1 was associated with a higher protozoa population in this diet (Table-7) as a host of methanogens that produce CH_4_ [45].

Supplementation with protein concentrates derived from fresh *Indigofera* leaves (IFW1 ration) potentially increases total VFA production by up to 33.56% compared with the control diet (69.44 vs. 52.07 mM), although this difference lacks statistical significance (Table-6). This suggests high variability in VFA production across different ration treatments. Although not statistically significant, the observed increase of 33.56% in total VFA production is noteworthy, highlighting the potential impact of protein concentrate supplementation from fresh *Indigofera* leaves. Total VFA production resulting from fresh *Indigofera* leaves in this study aligns with findings from other studies on an ammoniated rice straw-based diet supplemented with molasses or banana root plant meal resulting in 36.77–47.57 mM [46] and a combination of four levels of paddy straw (15%–30%) with alfalfa hay (25%–40%) which reported ranges 41.7–63.9 mM [47]. The partial VFAs, including C_2_, C_3_, and C_2_:C_3_ ratios, showed no significant differences (p > 0.05) in all diets due to the similar NDF content in the basal rations. In addition, the partial concentrations of iC_4_, iC_5_, and nC_5_ (Branch chain VFA [BCVFA]) in IFW1 and IFW05 were comparable to those in the control diet. However, despite the similar partial concentrations of iC_4_, iC_5_, and nC_5_ VFAs between IFW1, IFW05, and the control ratio, microbial protein production was higher in the former two (Table-7). This suggests that the BCAAs present in the LPC (Table-3) were deaminated to produce BCVFA, which were efficiently utilized for microbial protein synthesis.

The lower proportion of BCVFAs observed in IFN1 and IFN05 treatments is due to some of BCAA in this protein concentrate were not deaminated by rumen microbial. This phenomenon is likely a result of the use of 0.1 N NaOH solution during the extraction of LPC, which may have prevented protein degradation. A similar protein protection effect against rumen degradation was reported in sunflower protein extraction, where the use of NaOH resulted in increased undegradable rumen protein due to oxidation [48].

Protozoa represent half (50%) of the total microbial biomass in rumen, significantly contribute to anaerobic fermentation, and play a role in digesting the fiber derived from forage feed in ruminants [49]. Although the biological values of bacterial and protozoa proteins were considered similar, the digestibility of protozoa proteins was much greater than that of bacterial proteins [50, 51].

The present *in vitro* study demonstrated the importance of BCAA addition in low-quality basal diets to improve the rumen fermentation kinetics of ruminant animals. The promising results of the present study require further *in vivo* research to explore and determine the impact of LPC administration on increasing livestock productivity in ruminants. LPC from *Indigofera* can be a promising feed supplement for ruminants because it is widely distributed in tropical areas.

## Conclusion

The use of distilled water instead of NaOH to extract protein concentrates from fresh *Indigofeara* leaves produced the highest protein yield of 39.83%, accompanied by a notable increase in protein content (412.1 g/kg). Protein concentrates derived from fresh *Indigofera* increase the composition of branched AAs, including valine, leucine, and isoleucine, up to 52.17%, 61.84%, and 48.75%, respectively. These findings confirm the efficacy of distilled water as a solvent for optimizing protein extraction and enhancing the nutritional profile of fresh *Indigofera* leaves.

Supplementation of 1% protein concentrate extracted using distilled water from fresh *Indigofera* leaves into rice straw-based rations stimulated rumen fermentation, as demonstrated by a notable increase in total VFA and rumen microbial protein concentration. *In vitro* digestibility confirmed that protein concentrate extracted with distilled water from fresh *Indigofera* leaves resulted in higher gas production, total VFA, and microbial protein, while DM digestibility was similar to that of 0.1 N NaOH.

Findings from this study are the first to confirm that distilled water, as an environmentally friendly and cost-effective solvent, can successfully extract protein concentrate from fresh *Indigofera* leaves. This method provides an alternative solvent for evaluating foliage protein concentrates for livestock. Furthermore, this study demonstrated the potential of fresh *Indigofera* leaves to enhance rumen fermentation in low-story rice straw diets.

## Authors’ Contributions

WP: Conceptualization, methodology, investigation, data curation, formal analysis, drafted, edited, and reviewed the manuscript, validation, visualization, and supervision. DY: methodology, data curation, investigation, formal analysis, drafted, edited, and reviewed the manuscript, and visualization. TH: Conceptualization, methodology, investigation, data curation, formal analysis, and drafted the manuscript. SIWR: Conceptualization, investigation, formal analysis, and reviewed and edited the manuscript. YW: Methodology, data curation, and drafted and edited the manuscript. DAK: Formal analysis, validation, visualization, and drafted, edited, and reviewed the manuscript. EW: Conceptualization, formal analysis, and drafted, edited, and reviewed the manuscript. AJ: Validation and drafted and edited the manuscript. MA: Data interpretation, drafted, reviewed and edited the manuscript. All authors have read and approved the final manuscript.
